# An ethnobotanical study of medicinal plants used by local people in the lowlands of Konta Special Woreda, southern nations, nationalities and peoples regional state, Ethiopia

**DOI:** 10.1186/1746-4269-5-26

**Published:** 2009-09-24

**Authors:** Tesfaye Hailemariam Bekalo, Sebsebe Demissew Woodmatas, Zemede Asfaw Woldemariam

**Affiliations:** 1 Louis Dreyfus Commodities-Coffee Division, PO Box 1120 code 1250, Addis Ababa, Ethiopia; 2 Addis Ababa University, the National Herbarium (Department of Biology), PO Box 3434, Addis Ababa, Ethiopia

## Abstract

**Background:**

Research was carried out in Konta Special Woreda (District); it is a remote area with lack of infrastructure like road to make any research activities in the area. Therefore, this research was conducted to investigate medicinal plants of the Konta people and to document the local knowledge before environmental and cultural changes deplete the resources.

**Methods:**

The information was collected between October 2006 and February 2007. Interview-based field study constituted the main data collection method in which the gathering, preparation, use, previous and current status and cultivation practices were systematically investigated. The abundance, taxonomic diversity and distribution of medicinal plants were studied using ecological approach.

**Results:**

A total of 120 species, grouped within 100 genera and 47 families that are used in traditional medical practices were identified and studied. The Fabaceae and Lamiaceae were the most commonly reported medicinal plants with 16 (13.3%) and 14 (12%) species, respectively. 25.4% of the total medicinal plants are collected from homegardens and the rest (74.6%) are collected from wild habitats. Of the total number of medicinal plants, 108 species (90%) were used to treat human ailments, 6 (5%) for livestock diseases and the remaining 6 (5%) were used to treat both human and livestock health problems. The major threats to medicinal plants reported include harvesting medicinal plants for firewood (24.8%) followed by fire (22.3%) and construction (19%). Of the four plant communities identified in the wild, more medicinal plant species (34) were found in community type-4 (*Hyparrhenia cymbaria*-*Erythrina abyssinica *community), which accounted for 61.8%.

**Conclusion:**

Konta Special Woreda is an important area for medicinal plants and associated local knowledge; the natural vegetation being the most important reservoir for the majority of the medicinal plants. Environmental and cultural changes are in the process of threatening the resources and this signals the need for serious efforts to create public awareness so that measures are taken to conserve the medicinal plants in the natural ecosystems and other suitable environments.

## Introduction

Medicinal plants have important contributions in the healthcare system of local communities as the main source of medicine for the majority of the rural population. Plants have not only nutritional value but also, in the eyes of the local people, they have medicinal and ritual or magical values [1]. The ethnomedicinal healing systems vary across cultures. In Ethiopia, there is cultural diversity with various patterns of using the flora [2].

According to the World Health Organization (WHO), more than 3.5 billion people in the developing world rely on medicinal plants as components of their healthcare [3]. The vast majority of people (70-80%) in Africa consult Traditional Medical Practitioners (TMPs) for their healthcare [4]. Traditional medicine has been brought into focus for meeting the goals of a wider coverage of primary healthcare delivery, not only in Africa but also, in all countries of the world. It is the first choice healthcare treatment for at least 80% of Africans who suffer from high fever and other common ailments [5]. Thus, medicinal plants are widely used in the treatment of numerous human and livestock diseases in different parts of the world.

In Ethiopia, 80% of the people use medicinal plants and plant remedies selected over centuries. Moreover, medicinal plants remain the most important and sometimes the only source of therapeutics [6]. A study by Hamilton [7] attributed the dependence on medicinal plants to the low proportion of medical doctors to patients in Africa (Ethiopia 1:33,000; Kenya 1:7142; Tanzania 1:33,000; Uganda 1:25,000, Malawi 1:50,000; Mozambique 1:50,000; South Africa 1:1639; Swaziland 1:10,000).

Medicinal plants play a key role in the development and advancement of modern studies by serving as a starting point for the development of novelties in drugs [8]. The knowledge and use of plants is an integral part of many ethnic rural cultures in Ethiopia, the extent of which has not yet been studied in depth [1]. This study was carried out in the lowlands of Konta Special Woreda (District) to document the traditional knowledge of local peoples on medicinal plants (the ethnobotany); and to inevstigate the distribution, abundance and taxonomic diversity of medicinal plants. The medicinal plant lore and their distribution in the natural vegetation and homegardens have as yet not been studied in the Special Woreda.

## Materials and methods

### The Study Area

**Figure 1:**
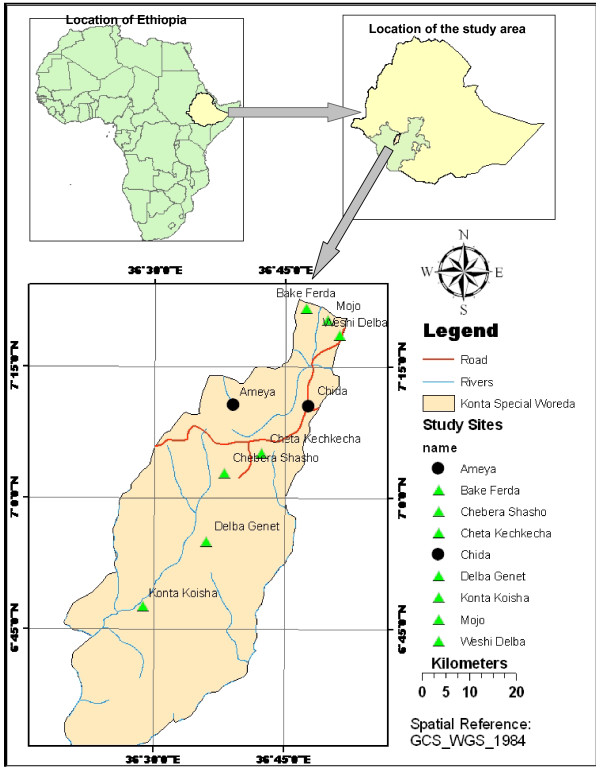
**Map of Konta Special Woreda showing the study Kebeles**.

Konta Special Woreda is located in South-Western Ethiopia in Southern Nations, Nationalities and Peoples Regional State (Figure 1), the special woreda is found between altitudinal ranges of 1200 and 1640 m.a.s.l and located between 6°30' N and 7°25' N, and 36°15' E and 36°55' E.

Based on the meteorological data recorded by the Ethiopian National Meteorological Service Agency for 6 years (January 2000 to December 2005), the study area had uni-modal rainfall distribution with the highest rain falling from July to September. The mean annual rainfall of the study area was 1374.1 mm, and the mean annual temperature was 22.98°C.

The Special Woreda is found between altitudinal ranges of 514 and 3305 m.a.s.l, and according to the broad vegetation types of Ethiopia [9], the vegetation of the Special Woreda is included in the *Combretum-Terminalia *Woodland, Savanna and Dry Evergreen Montane Forest and Grassland Complex. It is observed to be heterogeneous which includes short grassland, bushland and woodland. Of the total area of the Special Woreda (225,376 ha) the woodland vegetation covers (63,300 ha), trees planted through afforestation program (9 ha), bushland (10,509 ha), pastureland (24,517 ha), water body (60 ha), arableland (76,625 ha), perennial crops (4,830 ha), annual crops (30,103 ha), unproductive land (8,789 ha) and others (5,634 ha) (Socio-economic data of the Konta Special Woreda, 1994 (unpublished)).

The Konta Special Woreda has a population of 74,828 out of which the number of males and females are 36,128 and 38,700 respectively [10]. The three largest ethnic groups reported in the Special Woreda are Konta (86.19%), Kaffa (5.37%), Tsara (3.25%) and others (5.19%); and also Konta language is spoken as a first language by 85.14%, 6.71% Kaffa and 2.28% speak Tsara, the remaining 5.87% spoke other languages [11].

The economy of the local people is mainly based on subsistence agriculture where mixed farming is a common practice. They cultivate fruits, cereals (tef, maize and sorghum), spices and forest coffee. Moreover, they are engaged in rearing livestock including goats, sheep, donkeys, mules and chicken. The waste from livestock serves as organic fertilizer for homegarden plants. The average land per household in the Special Woreda is 2 ha (Socio-economic data of the Konta Special Woreda, 1994 (unpublished)).

### Methods

#### Ethnobotanical data collection

Ethnobotanical data were collected between October 2006 and January 2007, based mainly on semi-structured interviews with selected knowledgeable elders [12,13]. Information regarding the gathering, preparation, use, previous and current status and cultivation practice of medicinal plants were collected. Interviews and discussions were conducted in the Konta language, the common local language in the study area. A total of 70 knowledgeable elders (47 men and 23 women), between the ages of 20 and 80 (the majority in the age class of 41-50 years) were involved from the seven study *Kebeles *(the smallest administrative units in Ethiopia), taking ten from each *Kebele*. The *Kebeles *are Cheta-Kechkecha, Weshi-Delba, Mojo, Bake-Ferda, Delba-Genet, Chebera-Shaho and Konta-Koisha. The knowledgeable elders were selected with the help of local administraters, personnel in the Agriculture and Rural Development Office at each study *Kebele *and the local community. For ethical purpose data were collected with permission of the informants and knowledge of the local administration.

Preference ranking technique was used to investigate the degree of scarcity of some selected medicinal plants. In this exercise, each informant was asked to rank items based on a given criterion (in this case degree of scarcity of medicinal plants in the area) and their personal preference or perceived degree of importance [12]. The most important or preferred items (in this case the most scarce medicinal plants) were assigned the highest score (7), while the least preferred/abundant species was given the lowest (1). For this purpose, 10 individuals were randomly selected from the people that had already served as key informants. Each one of the informants was provided with fresh specimens of nine scarce medicinal plants, reported as scarce by most of the key informants, and asked to rank them according to their degree of scarcity.

Paired comparison method was used to determine the relative importance of plant species, which are used in the treatment of cough. In paired comparison, items are presented in pairs and decisions are made by individual respondents on the relative importance of one of the items from a pair [12]. In this case, five medicinal plants were paired with each other to be choosen by ten of the key informants. The total number of possible pairs (10) was obtained by applying the formula **n(n-1)/2**, where **n **is the number of medicinal plants being compared.

#### Vegetation data collection

Vegetation data were collected in order to classify and describe plant communities in the wild and plant associations in the homegarden, and to assess the distribution of the reported medicinal plants in the area. A total of 70 sample plots were established, 35 of which were in homegarden (five plots in each study *kebele*) and another 35 plots in the wild/outside homegardens (five plots in each study *kebele*). The size of the plots in homegardens were 5 × 5 m; in each homegarden three plots were taken from right, left and back side of the homegarden in order to assess the whole plant species. Then the averages of the three samples were considered for the data analysis. The plots taken in the natural vegetation were along a transect line, the size of the plot for tree species was 20 × 20 m [14]. Within the plots, plant number and species cover were estimated.

#### Plant identification

Voucher specimens were collected for each plant species encountered with the exception of some very common cultivated plants, which were identified in the field. Preliminary identification of the collected specimens was made in the field, then they were dried, deep-frozen and identified in the National Herbarium (ETH), Addis Ababa University using the published volumes of the Flora of Ethiopia and Eritrea [15-22] and by comparing with authentic herbarium specimens and finally confirmed by assistance of taxonomists. All voucher specimens were deposited at the National Herbarium.

### Data analysis and presentation

#### Ethnobotanical data analysis

Descriptive statistical method was employed to analyse and summarize the ethnobotanical data on the reported medicinal plants and associated knowledge. The relative importance of different plants in a given community was determined based on the consensus of informants' responses. It was calculated from the proportion of informants who independently reported knowledge on a given use against a particular disease or disease category following the approach used by Phillips and co-workers [23]. The informants' consensus was also used to investigate the effectiveness of medicinal plant/s to treat a particular ailment.

In preference ranking technique each informant was asked to rank items based on a given criterion. Then the numbers summed up for all respondents, giving an overall ranking for the objects by the selected groups of respondents [12]. Total rank of a paired comparison was obtained by summing the number of times each item was chosen. An item with the highest frequency of choices has the highest score. Responses of all selected informants were added to make general statements about the items [12].

#### Vegetation data analysis

The programme Biodiversity Professional, ver. 2 [24] was used to classify the vegetation into community and homegarden types based on the information obtained from the sample plots.

## Results

### Medicinal plants and associated indigenous knowledge

The informants reported 120 plant species that they use for medicinal purpose (Additional file 1). The plants belong to 100 genera and 47 families. Sixteen species belong to the family Fabaceae (13.3%) while the Lamiaceae and Asteraceae consisted fourteen (12%) and thirteen species (11%), respectively.

Of the 120 medicinal plants, 108 species (90%) are used against human ailments, six species (5%) are used to treat health problems of livestock and the remaining six species (5%) are used to treat both human and livestock ailments (Additional file 1 and table 1). The species used for both humans and livestock are *Setaria megaphylla*, *Pentas lanceolata*, *Justicia betonica*, *Phyllanthus sepialis*, *Calpurnia aurea *and *Solanum incanum*. In this study, 47 different humans and livestock health problems were encountered (39 in human and 8 in livestock).

**Table 1:** Medicinal plants used to treat livestock diseases

**Scientific name**	**Family**	**Voucher specimen no.**	**Local name**	**Habit**	**Application**	**part used**	**Method of preparation**	**Administration route**	**Livestock treated**
*Biophytum abyssinicum *A. Rich.	Oxalidaceae	THB-087	Denggo	Herb	To expel leech	Above ground	Fresh crushed above ground part homogenized in water adding salt	Oral	Cattle
*Calpurnia aurea *(Ait.) Benth.	Fabaceae		Cadhdhiw	Shrub	Snake bite	Root, Stem or Leaf	Any part of the plant crushed and homogenized in water to drink	Oral	Cattle
*Grewia mollis *Juss.	Tiliaceae	THB-051	Gumere	Tree	Constipation	Bark	Crushed fresh bark homogenized in water to drink adding salt	Oral	Cattle
*Justicia betonica *L.	Acanthaceae		Goppe dhaliyaa	Herb	Snake bite	Root	Fresh crushed root homogenized in water to drink	Oral	Cattle
*Pavetta abyssinica *Fresen.	Rubiaceae	THB-015	Bootha bekkaa	Shrub	*Mitch *& evil eye	Leaf	Crushed fresh leaf homogenized in water to drink and with the residue soak the whole body	Oral & Skin	Cattle
*Pentas lanceolata *(Forssk.) Defl. Subsp. *quartiniana *(A. Rich.) Verdc.	Rubiaceae		Mithaa	Herb	Snake bite	Root	Crushed fresh root homogenized in water to drink	Oral	Cattle
*Phyllanthus sepialis *Muell. Arg.	Euphorbiaceae		Cadhdho	Shrub	Snake bite	Root, Stem or Leaf	Any part of the plant crushed and homogenized in water to drink	Oral	Cattle
*Setaria megaphylla *(Steud.) Th. Dur. & Schinz	Poaceae		Daawaa	Herb	Snake bite	Leaf	Smell crushed fresh leaf then soak the whole body	Nasal & skin	Cattle
*Solanum dasyphyllum *Schumach.*	Solonaceae	THB-002	Buluwaa	Herb	Trypanosomiasi, cough	Fruit	Slices of fresh fruits homogenized in water adding salt	Oral	Cattle
*Solanum incanum *L.	Solonaceae		Buluwaa	Shrub	Cough	Fruit	The inner part of the fruit homogenized in water to drink	Oral	Cattle
*Syzygium guineense *subsp. *Macrocarpum *(Engl.) F. White	Myrtaceae	THB-169	Ocha	Tree	Diarrhea	Bark	Crushed fresh bark homogenized in water to drink	Oral	Cattle
*Tetradenia riparia *(Hochst. in C. Krauss) Codd*	Lamiaceae	THB-046	Hookko	Shrub	Diarrhoea, to improve milk content of cows	Leaf	Crushed fresh leaf homogenized in water and add salt	Oral	Cattle

Mitch: A disease of cattle with a common symptom of wound on the skin and make the hair remove easily

* Plants cultivated in homegardens with medicinal application

**Figure 2:**
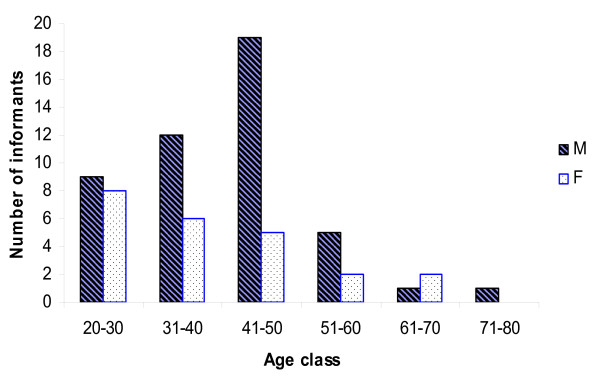
**The distribution of informants in age-classes**.

The distribution of informants in age class shows that, the majority of informants interviewed in this study were in the age class of 41-50 (Figure 2).

### Preparation, dosage and route of administration of medicinal plants

**Figure 3:**
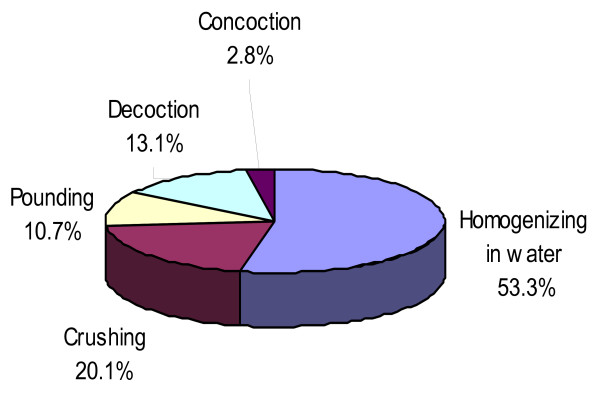
**Preparation methods of herbal medicine**.

Plant parts are prepared as medicine using fresh material (85.9%), dried plant material (9.5%) and combinations of these (4.6%) (Additional file 1 and table 1). Most of the plant remedies are prepared through the forms of homogenizing in water (53.3%) followed by crushing (20.1%), pounding (10.7%), decoction (13.1%) and concoction (2.8%) (Figure 3). Some specific herbal preparations are taken by mixing with food or drunk together with coffee prepared from the beans or leaves of the coffee plant (Additional file 1).

In the study area, the recommended dosage differs among informants for treating the same health problem. During the interview and discussion with the informants they indicated that the doses for liquid preparations were prescribed through estimation, in terms of a full, half or one fourth of a coffee cup, depending on the age of the patient being treated. Regarding some herbal preparations that are considered harmless, the dosage depends on the interest and/or the capacity of the patient to chew a particular plant for a given health problem (e.g. chewing the roots of *Cissampelos mucronata *to get relief from abdominal cramp).

With regards to method of application (route of administration), most medicinal plant preparations are taken internally (79.7%) out of which drinking takes the leading (67.8%) and externally (20.3%) soaking the medicinal preparation on the skin is the principal (38.3%) (Table 2).

**Table 2:** Method of application of medicinal plants in the study area

**Internal application**	**Total applications**	**% of total**	**External application**	**Total applications**	**% of total**
Drinking	160	67.8	Soaking	23	38.3
Chew & swallow the juice	31	13.1	Smearing	13	21.7
Dropping	15	6.4	Chew & spit the residue	10	16.7
Smelling	13	5.5	Tie on/hold on	8	13.3
Smoke bath inhalation	11	4.7	Rubbing/ointment	6	10
Eating mixing with food	5	2.1			
Steam bath inhalation	1	0.4			

**Total (for internal)**	**236**	**100**	**Total (for external)**	**60**	**100**

Internal application total	236	79.7			
External application total	60	20.3			

**Grand total**	**296**	**100**			

### Plant parts used for medicine and diversity of growth forms

**Figure 4:**
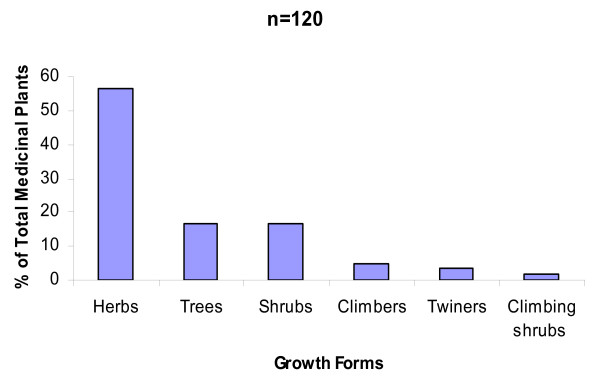
**Growth forms of medicinal plants**.

The interview result on different plant parts utilized revealed that leaves are the most commonly used and accounted for 34.2% of the total, followed by roots (30.9%), barks (8.2%) and other parts (19.8%). The analysis of growth forms shows that, of the total medicinal plants (n = 120) herbs were represented by 68 species (56.6%), trees and shrubs were represented by 20 species (16.7%) each (Figure 4).

### Habitat, current status and threats to medicinal plants

Informants in the study area confirmed that, medicinal plants are collected from different habitats. Of the total medicinal plants, 25.4% are collected from homegardens and the rest (74.6%) are collected from wild habitats (near farmland, forest, road side, around grazing land, close to streams/river, grassland and fallow land). It was noted that, only 25 species (20.8%) of the total 120 medicinal plants are under cultivation in homegardens (Additional file 1 and table 1, indicated using asterisks). Some of the medicinal plants (*Brassica juncea, Echinops kebericho *and *Nigella sativa*) are not occurring in the study area, but utilized by the people to treat different ailments by purchasing from markets when the need arises.

The results of interviews made with informants show that 7.7% of the medicinal plants are rarely encountered in the study area. The quantity of the medicinal plants was reported to have been decreasing from time to time. However, 92.3% of the respondents noted that medicinal plants that were cited are either commonly or occasionally found (Table 3). The majority of these are wild herbs that grow in different habitats such as fallow lands, grazing land, roadsides and in and around homegardens.

**Table 3:** Three categories of medicinal plants based on the degree of abundance as reported by informants

**Rarely encountered** **(7.7% of the total)**	**Occasionally encountered** **(31.6% of the total)**	**Commonly encountered** **(60.7% of the total)**
*Asparagus recemosus*	*Aerva lanata*	*Achyranthes aspera*
*Buddleja polystachya*	*Alysicarpus glumaceus*	*Acmella caulirhiza*
*Dicliptera laxata*	*Anarrhinum forskaohlii*	*Ajuga alba*
*Gnidia glauca*	*Biophytum abyssinicum*	*Allium cepa*
*Indigofera emarginella*	*Calpurnia aurea*	*Allium sativum*
*Pittosporum viridiflorum*	*Carissa spinarum*	*Artemisia absinthium*
*Plectranthus lanuginosus*	*Chamaecrista wittei*	*Artemisia abyssinica*
*Tetradenia riparia*	*Clematis simensis*	*Artemisia afra*
*Vepris dainellii*	*Cordia africana*	*Barleria ventricosa*
	*Crossandra nilotica*	*Carica papaya*
	*Croton macrostachyus*	*Centella asiatica*
	*Cyprus fischerianus*	*Cissampelos mucronata*
	*Dalbergia lactea*	*Citrus aurantifolia*
	*Desmodium adscendens*	*Clematis hirsuta*
	*Desmodium velutinum*	*Cleome gynandra*
	*Dombeya torrida*	*Clerodendrum umbellatum*
	*Erythrina abyssinica*	*Commelina latifolia*
	*Gardenia ternifolia*	*Conyza bonariensis*
	*Haumaniastrum villosum*	*Crotalaria hyssopifolia*
	*Indigofera spicata*	*Cuscuta campestris*
	*Justicia betonica*	*Cymbopogon citrates*
	*Lantana ukambensis*	*Cynoglossum coeruleum*
	*Lantana trifolia*	*Dichrocephala integrifolia*
	*Lepidium sativum*	*Dissotis canescens*
	*Lysimachia ruhmeriana*	*Dracaena steudneri*
	*Moringa stenopetala*	*Dyschoriste multicaulis*
	*Mucuna melanocarpa*	*Erythrina brucei*
	*Paullinia pinnata*	*Eucalyptus camaldulensis*
	*Phyllanthus sepialis*	*Euphorbia schimperiana*
	*Piper capense*	*Ficus sur*
	*Polygala sadebeckiana*	*Ficus thonningii*
	*Pterolobium stellatum*	*Grewia mollis*
	*Sanicula elata*	*Guizotia abyssinica*
	*Sapium ellipticum*	*Indigofera arrecta*
	*Spermacoce sphaerostigma*	*Indigofera garckeana*
	*Thunbergia ruspolii*	*Indigofera zenkeri*
	*Tragia brevipes*	*Isodon ramosissimus*
		*Ipomoea purpurea*
		*Kyllinga bulbosa*
		*Lactuca inermis*
		*Laggera pterodonata*
		*Leonotis ocymifolia*
		*Leucas stachydiformis*
		*Leucas deflexa*
		*Monopsis stellarioides*
		*Musa paradisiaca*
		*Ocimum basilicum Var. thyrsiflorum*
		*Ocimum americanum*
		*Ocimum gratissimum*
		*Ocimum lamiifolium*
		*Oldenlandia goreensis*
		*Oxalis corniculata*
		*Pavetta abyssinica*
		*Pentas lanceolata*
		*Phytolacca dodecandra*
		*Plectranthus ornatus*
		*Ricinus communis*
		*Rumex nepalensis*
		*Ruta chalepensis*
		*Setaria megaphylla*
		*Sida rhombifolia*
		*Solanum dasyphyllum*
		*Solanum incanum*
		*Sonchus bipontini*
		*Sonchus oleraceus*
		*Stephania abyssinica*
		*Syzygium guineense subsp.*
		*Macrocarpum*
		*Terminalia schimperiana*
		*Thalictrum rhynchocarpon*
		*Tragia doryoides*
		*Vernonia amygdalina*

The threats to medicinal plants reported include harvesting medicinal plants for firewood (24.8%) followed by fire (22.3%), construction (19%), charcoal (12%), agricultural expansion & shifting cultivation (10.4%) and others (11.5%). Due to frequent fires in the dry season the medicinal plants are being lost from the area, making it difficult to find easily during data collection period.

### Degree of Scarcity of Medicinal Plants

Preference ranking of nine scarce medicinal plants that were reported as being rare conducted to analyse the degree of scarcity of medicinal plants as shown in Table 4, *Vepris dainellii *scored the highest mark and ranked first indicating that it was the most scarce followed by *Pittosporum viridiflorum *and *Tetradenia riparia*.

**Table 4:** Preference ranking values of nine selected medicinal plants by key informants based on degree of scarcity

**List of medicinal plants**	**Key informants (coded A to J) with the ranks they gave**										**Total score**	**Rank**
			
	**A**	**B**	**C**	**D**	**E**	**F**	**G**	**H**	**I**	**J**		
*Asparagus recemosus*	1	1	3	6	7	2	4	7	3	7	41	5
*Buddleja polystachya*	5	7	4	4	4	3	5	5	1	1	39	7
*Dicliptera laxata*	7	5	3	1	3	1	1	3	2	2	28	9
*Gnidia glauca*	1	3	7	2	2	7	2	1	6	6	37	8
*Indigofera emarginella*	3	2	2	5	7	5	5	5	6	7	47	4
*Pittosporum viridiflorum*	7	7	5	6	5	6	7	4	4	3	54	2
*Plectranthus lanuginosus*	4	4	6	3	1	6	3	2	7	4	40	6
*Tetradenia riparia*	2	6	1	7	6	4	4	6	7	6	49	3
*Vepris dainellii*	6	7	5	6	6	7	6	7	5	5	60	1

**Key**-Scores in the table indicate ranks given to medicinal plants based on their scarcity. Highest number (7) for the medicinal plant which informants thought most scarce in the area and the lowest number (1) for the least scarce medicinal plant.

### Medicinal plants used to treat a particular and several ailments

The degree of agreement between informants on each medicinal plant and the popularity of some medicinal plants in treating specific health problem was examined in detail. *Croton macrostachyus *stood as the most popular medicinal plant having been cited by twelve out of the total of 70 informants (17.1%) for its medicinal value to treat wound, followed by *Pentas lanceolata *mentioned by eight informants (11.4%) as medicine for snakebite, *Rumex nepalensis *and *Vepris dainellii *mentioned by seven (10%) informants each for treating abdominal cramp. The other reported remedies such as, *Indigofera spicata*, *Moringa stenopetala *and *Phytolacca dodecandra *were mentioned by six (8.6%) informants each and *Buddleja polystachya *was mentioned by five (7.1%) informants indicating that these medicinal plants are well known within the community for their effectiveness regarding specific health problem. Some species are used to treat many ailments (Additional file 1 and table 1).

### Medicinal plants and trade

In the study area, it is very rare to see medicinal plants in the market. Only some species such as *Artemisia absinthium, A. abyssinica, A. afra, Brassica juncea, Ocimum basilicum var. thyrsiflorum, Echinops kebericho *and *Lepidium sativum *are sold in the market for their medicinal value. During the interview, informants mentioned that, fresh seeds of *Vepris dainellii *were also sold in the local market. Some other medicinal plants that were also marketed for other use-values as spices and fruits include: *Allium cepa, A. sativum, Carica papaya, Citrus aurantifolia, Cymbopogon citratus, Plectranthus ornatus, Nigella sativa, Lepidium sativum *and *Ruta chalepensis*.

### Importance of medicinal plants in the study area

Results of pairwise comparison made to determine the most preferred medicinal plants among the five species that were used to treat cough using ten key informants showed that *Ocimum americanum *stood first followed by *Eucalyptus camaldulensis *(Table 5). *Ocimum americanum *is the most favored species while *Artemisia afra and Ocimum basilicum *var. *thyrsiflorum *are the least favoured over the other plant species cited in treating cough.

**Table 5:** Results of pairwise comparison of medicinal plants used for treating cough

**Plant species**	**Scores given by Respondents (R_1_-R_10_)**										**Total Score**	**Rank**
			
	**R**_1_	**R**_2_	**R**_3_	**R**_4_	**R**_5_	**R**_6_	**R**_7_	**R**_8_	**R**_9_	**R**_10_		
*Artemisia abyssinica*	2	2	1	1	2	0	4	2	3	2	19	3^rd^
*Artemisia afra*	4	1	3	3	1	1	0	1	2	1	17	4^th^
*Eucalyptus camaldulensis*	3	1	1	3	3	2	2	0	2	3	20	2^nd^
*Ocimum americanum*	1	4	2	2	2	4	1	4	3	4	27	1^st^
*Ocimum basilicum *var. *thyrsiflorum*	0	2	3	1	2	3	3	3	0	0	17	4^th^

### Distribution of medicinal plants in plant community types outside homegardens

The majority of medicinal plants (74.4%) that are collected in the study area were from outside the homegarden, distributed in different community types. A total of 138 plant species were recorded from 35 sample plots. Based on the cover abundance data of species, the sample plots were classified into four community types (1-4). Each community is named by a combination of the characteristic species, which have the highest mean cover abundance values. The medicinal plants encountered in each of the four community types are given in Table 6.

**Table 6:** Plant community types and medicinal plants found in them

**Plant Community Type**	**No of MPS**	**List of Medicinal Plant Species (MPS)**
*1 Cordia Africana *-*Maesa lanceolata *community	21	*Cordia africana, Phytolacca dodecandra, Erythrina brucei, Ficus sur, Grewia mollis, Dracaena steudneri, Croton macrostachyus, Eucalyptus camaldulensis, Ricinus communis, Indigofera arrecta, Euphorbia schimperiana, Conyza bonariensis, Syzygium guineense, Sonchus oleraceus, Leonotis ocymifolia, Achyranthes aspera, Solanum incanum, Sonchus bipontini, Leucas deflexa, Sida rhombifolia, Commelina latifolia*
*2. Sapium ellipticum *-*Ficus sur *community	18	*Sapium ellipticum, Ficus sur, Eucalyptus camaldulensis, Achyranthes aspera, Croton macrostachyus, Cuscuta campestris, Ficus thonningii, Phytolacca dodecandra, Cynoglossum coeruleum*, *Oldenlandia goreensis, Indigofera arrecta, Solanum incanum, Sonchus bipontini*, *Ricinus communis*, *Euphorbia schimperiana, Leucas deflexa, Sida schimperiana, Sida rhombifolia*
*3. Vitex doniana *-*Kyllinga bulbosa *community	12	*Kyllinga bulbosa, Gardenia ternifolia, Centella asiatica, Guizotia schimperi, Solanum incanum, Ocimum gratissimum, Eucalyptus camaldulensis, Triumfetta rhomboidea, Conyza bonariensis, Sonchus oleraceus, Sida rhombifolia, Cynoglossum coeruleum*
*4. Hyparrhenia cymbaria*-*Erythrina abyssinica *community	34	*Erythrina abyssinica, Cordia africana, Indigofera arrecta, Leonotis ocymifolia, Terminalia schimperiana, Sanicula elata, Ficus sur, Erythrina brucei, Clerodendrum umbellatum, Vernonia amygdalina, Clematis hirsuta, Conyza bonariensis, Guizotia schimperi, Leucas stachydiformis, Triumfetta rhomboidea, Sida rhombifolia, Solanum incanum, Syzygium guineense, Pentas lanceolata, Gardenia ternifolia, Centella asiatica, Cynoglossum coeruleum, Indigofera zenkeri, Justicia betonica, Ocimum gratissimum, Carissa spinarum, Lantana trifolia, Biophytum abyssinicum, Dalbergia lactea, Tephrosia elata, Setaria megaphylla, Justicia betonica, Leucas deflexa, Grewia mollis*

#### 1. *Cordia africana-Maesa lanceolata* community

This type of community was found at 1500 m.a.s.l in two of the seven sites (Cheta-Kechkecha and Bake-Ferda).

#### 2. *Sapium ellipticum-Ficus sur* community

This type of community was found at altitudes between 1200 and 1490 m.a.s.l. in four of the seven sites (Mojo, Chebera-Shaso, Delba-Genet and Konta-Koisha). The plots describing this community occur around gorges and riverine vegetation and constituted of different growth forms including trees (*Millettia ferruginea, Bridelia micrantha, Phoenix reclinata, Mimusops kummel*) and shrubs (*Maesa lanceolata, Coffea arabica, Senna septemtrionalis, Vernonia hymenolepis*).

#### 3. *Vitex doniana-Kyllinga bulbosa* community

This type of community was found at altitudes between 1200 and 1470 m.a.s.l. in three of the seven sites (Weshi-Delba, Chebera-Shasho and Delba-Genet). This is grassland vegetation serving for grazing purposes.

#### 4. *Hyparrhenia cymbaria-Erythrina abyssinica* community

This type of community was found at altitudes between 1200 and 1450 m.a.s.l. in two of the seven sites (Delba-Genet and Konta-Koisha). This community is also grassland vegetation with many different types of medicinal plants.

### Distribution of medicinal plants in the plant association of homegardens

Some of the medicinal plants (25.4%) that were collected in the study area were from the homegardens being distributed in different plant community types identified in homegardens. A total of 101 plant species were recorded from 35 sample plots taken in homegardens. Based on the cover abundance data of the species, the sample plots were classified into two homegarden plant associations (1 & 2). Each association is named by a combination of the characteristic species with the highest mean cover abundance value.

#### 1. *Colocasia esculenta - Ensete ventricosum - Coffea arabica* homegarden

*Colocasia esculenta, Ensete ventricosum *and *Coffea arabica *are the characteristic species of this type of homegardens. These types of homegardens were found at 1450 m.a.s.l. in one of the seven sites (Delba-Genet) and consisted of medicinal plants such as *Cordia africana, Ricinus communis, Rumex nepalensis, Sida rhombifolia, Artemisia abyssinica, Triumfetta rhomboidea, Cynoglossum coeruleum, Leucas deflexa *and *Cleome gynandra*.

#### 2. *Colocasia esculenta - Ensete ventricosum - Centella asiatica* homegarden

*Colocasia esculenta, Ensete ventricosum *and *Centella asiatica *are the characteristic species of this type of homegardens. These types of homegaredens were found at altitudes between 1200 and 1500 m.a.s.l. in six of the seven sites (Mojo, Chebera-Shaso, Weshi-Delba, Cheta-Kechkecha and Bake-Ferda and Konta-Koisha). The medicinal plants found in this type of homegaredens are *Centella asiatica, Commelina latifolia, Musa paradisiaca, Carica papaya, Sida rhombifolia, Achyranthes aspera, Guizotia schimperiana, Oxalis corniculata, Leucas deflexa, Setaria megaphylla, Justicia anagalloides, Acmella caulirhiza, Indigofera arrecta, Triumfetta rhomboidea, Ricinus communis, Cordia africana, Solanum incanum, Eucalyptus camaldulensis, Conyza bonariensis, Cynoglossum coeruleum, Ruta chalepensis, Vernonia amygdalina, Plectranthus ornatus, Erythrina brucei, Solanum dasyphyllum, Dichrocephala integrifolia, Artemisia afra, Artemisia abyssinica, Ocimum gratissimum, Cuscuta campestris, Cleome gynandra, Euphorbia schimperiana, Moringa stenopetala, Ocimum basilicum Var. thyrsiflorum, Artemisia absinthium, Cyprus fischeranus, Cymbopogon citratus, Cissampelos mucronata, Ocimum lamiifolium, Laggera pterodonata, Lepidium sativum, Clerodendrum umbellatum *and *Rumex nepalensis*.

This analysis revealed more communities of natural vegetation as compared to homegarden types. This indicates that, the vegetation outside cultivated landscapes has less interference by human activities and hence holds more plant species and associations. On the other hand, a small number of communities are obtained in homegardens due to the activities of the society; they cultivate plants based on their interests for different use-values and consequently there is a higher similarity of plant species within different plots of the homegardens.

## Discussion

### Medicinal plants and associated knowledge

The number of reported medicinal plants (120 species) and their uses by the community demonstrates the depth of the local indigenous knowledge on medicinal plants and their applications. Similar study undertaken in Bolivia came up with 129 plant species of medicinal importance [25] while in Taounate Province of Northern Morocco 102 species were discovered [26]. The study in Palestine likewise produced 165 species of medicinal plants [27]. On the other hand, the study in the region of Pallars came up with a large number of medicinal plants, about 437 species [28]. In general, various studies have shown that different areas in different parts of the world demonstrated the existence of considerable amount of indigenous ethnomedicinal knowledge.

The finding of the family Fabaceae as the contributor of higher number of species used for medicinal purposes than other families is in agreement with similar studies elsewhere in Ethiopia [29,2,30] and other country in Africa [31].

This study revealed that, most of the knowledge on herbal remedies is handled down to the younger members of the community by elders, who are 41-50 years old. This hints at the fact that ethnomedicinal knowledge is concentrated in the elderly members of the community and the relative difficulty in its transfer from the elders to the young generation. This might be related to the waning of interest of the young generation on indigenous knowledge. Different studies in different areas showed that medicinal plant knowledge and transfer of knowledge to the young generation have been affected by modernization (having access to modern education and health service) and environmental change [32-34].

Most of the medicinal plants (25%) recorded in this study are also medicinally useful in other parts of Ethiopia [35][1][36,33,37,34,2,30] and 12.5% of the medicinal plants are also recorded in other parts of the world 31[38-41][25][42] .

Some of the plants that were reported by the informants in the study area (*Solanum incanum, Thunbergia ruspolii, Buddleja polystachya, Citrus aurantifolia, Erythrina abyssinica, Pittosporum viridiflorum, Piper capense, Rumex nepalensis, Artemisia absinthium, Ocimum lamiifolium, Phytolacca dodecandra, Ruta chalepensis, Leucas deflexa *and *Carica papaya*) are also used for similar health problems in some parts of the country and elsewhere [35,1,33,38,37][2,39][25]. The fact that some of the reported plants are having similar uses elsewhere can be taken as indication of their pharmacological effectiveness having been tested in different areas by different cultures.

### Preparation, dosage and route of administration of medicinal plants

About 85.9% of the remedies were prepared from fresh plant materials in the study area, which is a common observation of different studies on various health problems [33,25]. Informants confirmed that dried and fresh medicinal parts are used at the same time for some health problems such as the treatment for snakebite where fresh root of *Indigofera zenkeri *is chewed and then dried stem and leaf are burnt and the smoke is used for smokebath treatment.

The results of this study showed that using concoction (2.8%) of plants for a given ailment is not a common practice. In different parts of the country using a single plant for a given health problem is common [33,29] and other investigations showed that most diseases and pains are usually treated with a single plant [25]. However, for serious health problems mixtures of different plants are used. There is a wide belief on the synergic effect of one plant on the other during the use of concoctions [43]. Kani tribals in India are said to usually prepare medicines from a combination of several plants as they believed that combinations of several plant parts cure diseases rapidly [40].

The present study revealed that different forms of preparations are investigated; some of them are homogenizing in water, crushing, pounding, decoction and concoction but homogenizing in water takes the lead [2]. A similar study showed that different preparations and application methods of medicinal plants were mentioned for internal and external use [42] in which water is mostly used to dilute plant preparations while some remedies are prepared from dry and fresh plant parts [33].

There is no standardized measure on the dose of herbal remedies in the study area; the dose depends on the herbalist that prepares the herbs for medicinal purpose. For example, the same plant species with specific part is recommended in different doses to treat similar ailments. Lack of standardization and quality control is seen as one of the main disadvantages of traditional medicine as summarized from various sources [44]. There is also lack of agreement among the informants on doses of certain remedies prescribed [33] and lack of precision on the dose. Various authors mention that one of the constraints of traditional medicinal plants is lack of appropriate dose for a given ailment. Moreover, the dose differs according to different cultures [45,2]. The toxicity of some medicinal plants and their potential to do harm is a common complaint among those who would like traditional medicine to be standardized. It is commonly believed that traditional practitioners either do not know the strength of their own medicines or do not bother to fit doses to the size or body weight of the patients [46]. However, it is known that some traditional healers do give different dosages and frequency of application depending on age, sex and other condition or vary the medicine itself on such differences.

In this study internal application takes the upper hand of which drinking is the most common mode of administration (67.8%). A study conducted in Bolivia shows that, the most frequently used route of administration is oral ingestion, which accounts for 57.5% [25]. The study on the Zay people (Central Ethiopia) showed that most of the remedies are taken orally, accounting for 26 (79%) species of the reported medicinal plants [33]. In another study [28], it was shown that most of the plants were used internally (68%) and only 32% being used for external application. Furthermore, most of the remedies were given orally, which was recorded for 52%, and the external application accounting for 35% of the medicinal preparations [34] while another [31] indicated that oral administration takes the largest part, about 80%.

### Plant parts used for medicinal purpose and the diversity of growth forms

Leaf is highly used for medicinal purpose (34.2%) than the other plant parts in the study area. Many studies conducted in different parts of Ethiopia and in many parts of the world also showed that leaves are used more than the other parts of a plant [36,33,29,39,40,30]. This practice helps to reduce the rate of threat on plant species or helps for sustainable harvesting of plants since removal of an appreciable amount of leaf is tolerated by the plant [33,40].

The present study showed that the Konta people use more of herbs (about 68 species) than trees (20 species) in a similar pattern as reported from India, where about 19 out of 54 species were herbs and shrubs were about 12 species [40]. More than half of the Zay plant remedies were obtained from herbs partly because forests have been degraded and that the informants affirmed that it takes much time and effort to harvest plant material from medicinal trees [33]. The trend of using more of herbaceous plants could be advantageous as it is easier to cultivate them when they are in short supply. Naturally, there are more herbaceous plant species as compared to trees.

### Habitat, current status and threats to medicinal plants

In the study area, about 74.6% medicinal plants were collected from wild habitat while the rest (25.4%) were collected from homegardens. Most of the medicinal plants (82%) which were utilized by the Zay people were harvested from the wild [33] while in another study [30] the majority (85.71%) of the cited medicinal plant species was collected from wild areas.

Wild habitats are subjected to the loss of a number of plant species due to different anthropogenic factors such as firewood collection (24.8%); frequent fire (22.3%) and harvesting medicinal plants for use in construction (19%). A study conducted in Sekoru District [30] showed that, there are different threats to medicinal plants such as deforestation (40%), drought (17.5%), agricultural expansion (12.5%) and fire (12.5%).

### Informant consensus

Informant consensus values give good indication about particular species that serve for particular health problems and about specific medicinal plants used for several health problems. Such information underlines the pharmacological significance of the medicinal plants in the area. Medicinal plants with higher informant consensus need to be seriously considered for further ethnopharmacological studies, since they are species widely applied by many people and they have been utilized for a long time [25].

### Medicinal plants and trade

Though harvesting medicinal plants for commercializing is not a common practice in the study area, there were some fresh collections marketed within the local community (e.g. *Artemisia afra, Plectranthus ornatus, Ocimum basilicum *var. *thyrsiflorum, Ruta chalepensis, Vepris dainellii *and *Echinops kebericho *(dry root)). In addition, parts of different medicinal plants are also marketed for their medicinal value and for use as spice (*Allium cepa, Allium sativum, Lepidium sativum *and *Nigella sativa*). Given the fact that most of these are cultivated species, commercialization does not have devastating effect on the survival of the plants. Nevertheless, it has been indicated that unsustainable harvesting of medicinal plants for commercial purposes has exerted excessive pressure on several species (eg. *Warburgia salutaris*) in Maputo, Mozambique [47]. Furthermore, the majority of medicinal plants commercialized in different African countries are trees and shrubs than herbs [4,48]. Since woody species take longer time to establish and grow to maturity, concern about conservation of medicinal plants is growing. Unsustainable harvesting of medicinal plants is threatening their genetic and species diversity and further affects the ecological stability of the habitats in which they are found.

### Plant associations in and outside homegardens

The four distinct plant community types obtained in the wild vegetation contrast with the two homegardens types. This indicates that, the wild vegetation reflects the natural distribution of plants with no major human influence in contrast with plants in homegardens which reflect the human management and decision making. Higher similarity of plant species are seen within different plots of the homegardens. Perhaps, it would be more appropriate to consider the plant communities identified in homegardens as homegarden types rather than community types, which is usually applicable to the natural vegetation. A study conducted in southern Ethiopia applied vegetation classification approaches to homegardens and identified community types [36], which could be interpreted as homegarden plant associations. Another study conducted in Wolayta and Gurage homegardens indicated that farmers apply elaborate criteria to assign crops to particular areas within their homegardens [49] again showing that farmer decision making is greatly different from the natural distribution (be it stochastic or deterministic causation).

## Conclusion

The results of the study revealed that there is high diversity of medicinal plants and traditional knowledge about the use, preparation, and application which is still maintained among the Konta people. The preservation of this knowledge appears to be the result of continued reliance of the local communities on the medicinal plants. However, the knowledge on herbal remedies is held by elders, who were between 41-50 years of age. The decline in the use of medicinal plants by younger generation may gradually lead to the fading away of the indigenous knowledge associated with the plants. Utilization of more herbs than trees and shrubs for medicinal purpose may hint at the fact that the pressure due to harvesting medicines is insignificant on plant diversity in the area. Again, using more leaves than other plant parts implies that traditional medical culture in the area does not threaten biological diversity. The results also revealed that many wild species are under growing pressures from various anthropogenic factors. Thus, public awareness and community based management need to be encouraged at all levels to maintain the biodiversity and the ethnomedicinal knowledge of the Konta people.

## Competing interests

The authors declare that they have no competing interests.

## Authors' contributions

THB identified the research area and title, collected field data, carried out statistical analysis and drafted the manuscript. SDW and ZAW participated in refining the title, formulation of the research problem, data analysis and drafting as well as enrichment of the manuscript. All authors took part in approving the final manuscript.

## Supplementary Material

Additional file 1 **Medicinal plants used for the treatment of human diseases**. The file lists plant species used to treat human ailments, scientific and local name of plant species, plant part used, voucher number, methods of preparation and application. Click here for file
