# Supplementary material for: An ethnobotanical study of medicinal plants used by local people in the lowlands of Konta Special Woreda, southern nations, nationalities and peoples regional state, Ethiopia
Source: J Ethnobiol Ethnomed. 2009 Sep 24;5:26. doi: 10.1186/1746-4269-5-26 (PMC2764637; doi:10.1186/1746-4269-5-26)
Supplement: Additional file 1 — Medicinal plants used for the treatment of human diseases. The file lists plant species used to treat human ailments, scientific and local name of plant species, plant part used, voucher number, methods of preparation and application. [file 1746-4269-5-26-S1.doc]

**Appendices**

Appendix 1. Medicinal plants used to treat human ailments in the study area

| **Scientific name** | **Family** | **Voucher specimen no.** | **Local name** | **Habit** | **Application** | **part used** | **Method of preparation** | **Administration route** |
| --- | --- | --- | --- | --- | --- | --- | --- | --- |
| *Achyranthes aspera* L. | Amaranthaceae | THB-013 | Zanggiya | Herb | - | - | - | - |
| *Acmella caulirhiza* Del. | Asteraceae | THB-038 | Aydaamiyaa | Herb | Abdominal distention | Flower & Leaf | Fresh flower and leaf crushed then homogenized in water to drink | Oral |
|  |  |  |  |  | Wound in mouth | Flower | Chewing fresh flower | Oral |
| *Aerva lanata* (L.) Juss. ex Schultes | Amaranthaceae | THB-066 | Picppiccaa | Herb | Constipation | Aboveground | Drinking decocted aboveground part of the plant | Oral |
| *Ajuga alba* (Guerke) Robyns | Lamiaceae | THB-041 | Kaare dhaliyaa | Herb | Diarrhea | Leaf | Crushed fresh leaf homogenized in water to drink | Oral |
| *Allium cepa* L.* | Alliaceae | - | Zo7o Shunkkurttiya | Herb | - | - | - | - |
| *Allium sativum* L.* | Alliaceae | - | Tuummuwaa | Herb | - | - | - | - |
| *Alysicarpus glumaceus* (Vahl.) DC. | Fabaceae | THB-058 | Qirccantte dhale | Herb | Snake bite | Root | Pounded dried root mixed with water drink | Oral |
|  |  |  |  |  | Evil eye | Leaf | Fresh leaf tied with cloth to put on neck like a necklace | Tie on |
|  |  |  |  |  |  | Root | Crushed fresh root homogenized in water to drink | Oral |

Appendix 1. Continued…

| **Scientific name** | **Family** | **Voucher specimen no.** | **Local name** | **Habit** | **Application** | **part used** | **Method of preparation** | **Administration route** |
| --- | --- | --- | --- | --- | --- | --- | --- | --- |
| *Anarrhinum forskaohlii* (Gmel.) Cufod. subsp. *abyssinicum* (Jaub. & Spach) D. A. Sutton | Scrophulariaceae | THB-062 | Woraa haso dhalee | Herb | Snake bite | Root | Chewing dry or fresh root or crushed fresh leaf homogenized in water to drink | Oral |
| *Artemisia absinthium* L.* | Asteraceae | THB-024 | Naattiruwaa | Herb | Abdominal cramp | Leaf | Crushed fresh leaf concocted with *Artemisia afra* and *Cuscuta campestris* | Oral |
|  |  |  |  |  | Abdominal distention, intestinal parasite | Leaf | A patient will drink crushed fresh leaf which is homogenized in water | Oral |
|  |  |  |  |  | Intestinal parasite | Leaf | Fresh crushed concoction of *Artemisia afra* and *Ruta chalepensis* homogenized in water to drink | Oral |
|  |  |  |  |  |  |  | Fresh crushed leave concocted with *Artemisia afra* and homogenized in water to drink | Oral |
| *Artemisia abyssinica* Sch. Bip. ex A. Rich.* | Asteraceae | THB-030 | Bushadhdhiiya | Herb | Evil spirit | Leaf | Crushed fresh root homogenized in water and the patient will drink, and the patient allowed smelling the residue. | Oral & Nasal |
|  |  |  |  | Herb | Cough | Leaf | Drink decocted fresh leaf mixing with butter | Oral |

Appendix 1. Continued…

| **Scientific name** | **Family** | **Voucher specimen no.** | **Local name** | **Habit** | **Application** | **part used** | **Method of preparation** | **Administration route** |
| --- | --- | --- | --- | --- | --- | --- | --- | --- |
| *Artemisia afra* Jacq. ex willd.* | Asteraceae | THB-019 | Aguppiyaa | Herb | Cough & to maintain health of infants | Leaf | Drink decocted fresh leaf mixing with butter | Oral |
| *Asparagus recemosus* Willd. | Asparagaceae | THB-161 | Zulua hargia dhale | Climbing Shrub | Liver disease | Root  Leaf | Crushed fresh root is homogenized in water to drink  On the next day crushed fresh leaf homogenized in water to drink | Oral  Oral |
| *Barleria ventricosa* Hochst. ex Nees | Acanthaceae | THB-007 | Gingginoo | Herb | Evil eye | Root | Crushed fresh root concocted with the leaf of *Leonotis ocymifolia* then homogenized in water to drink | Oral |
| *Brassica juncea* (L.) Czern. & Coss. | Brassicaceae | - | Sannaafiya | Herb | Pneumonia | Seed | Dried seed pounded with dried seed of *Lepidium sativum* & *Nigella sativa* with fresh bulb of *Allium sativum* then the concoction mixed with butter to drink | Oral |
| *Buddleja polystachya* Fresen. | Loganiaceae | THB-016 | Kamppaaraa | Shrub | Malaria | Leaf | Juice of fresh crushed leaf droped in nose | Nasal |

Appendix 1. Continued…

| **Scientific name** | **Family** | **Voucher specimen no.** | **Local name** | **Habit** | **Application** | **part used** | **Method of preparation** | **Administration route** |
| --- | --- | --- | --- | --- | --- | --- | --- | --- |
| *Calpurnia aurea* (Ait.) Benth. | Fabaceae | THB-053 | Cadhdhiw | Shrub | Toothache | Root | Crushed fresh root tied with cloth and put in tooth | Oral |
|  |  |  |  |  | Snake bite | Root, Stem or Leaf | Any part of the plant crushed and homogenized in water to drink | Oral |
| *Carica papaya* L.* | Caricaceae | THB-213 | Paappa | Tree | Intestinal parasite | Seed | Pounding dry seed then eat with food mixing with butter (if available) | Oral |
|  |  |  |  |  | Intestinal parasite | Root | Crushed fresh root homogenized in water to drink | Oral |
|  |  |  |  |  | Malaria | Leaf | Crushed fresh leaf homogenized in water to drink | Oral |
| *Carissa spinarum* L. | Apocynaceae | THB-075 | Laade/Algaa | Shrub | Snake bite | Root | Crushed or pounded fresh or dried root homogenized in water to drink  and Wash the bite with crushed fresh root | Oral  Skin |
| *Centella asiatica* (L.) Urban | Apiaceae | THB-011 | Ecare haythaa | Herb | Wound | Leaf | Juice of fresh crushed leaf dropped on the wound | Skin |
|  |  |  |  |  | Eye disease | Leaf | Crushed fresh leaf homogenized in water then the juice drop in eye | Eye |
| *Chamaecrista wittei* (Ghesq.) Lock | Fabaceae | THB-073 | Giicinddo | Shrub | Snake bite | Root | Chewing fresh root or crushed fresh root mixed with coffee prepared from matured leaf of coffee to drink | Oral |

Appendix 1. Continued…

| **Scientific name** | **Family** | **Voucher specimen no.** | **Local name** | **Habit** | **Application** | **part used** | **Method of preparation** | **Administration route** |
| --- | --- | --- | --- | --- | --- | --- | --- | --- |
| *Chamaecrista wittei* (Ghesq.) Lock | Fabaceae | THB-073 | Giicinddo | Shrub | Snake bite | Root | Crushed or pounded fresh or dried root homogenized in water to drink | Oral |
|  |  |  |  |  |  |  | Wash the bite with crushed fresh root | Skin |
|  |  |  |  |  |  |  | Smoke bath of fresh or dried root | Nasal |
| *Cissampelos mucronata* A. Rich. | Menisspermaceae | THB-079 | Bootha turaa | Climber | Abdominal cramp, to facilitate digestion | Root | Crushed fresh root homogenized in water to drink or chewing fresh root | Oral |
|  |  |  |  |  | Snake bite | Root | Crushed/pounded fresh/dried root homogenized in water to drink | Oral |
| *Citrus aurantifolia* (Christm.) Swingle* | Rutaceae | THB-032 | Loomiyaa | Tree | Scabies | Fruit | Washing the diseased part with infused leaf of *Vernonia amygdalina* and *Ocimum lamiifolium* | Skin |
| *Clematis hirsuta* Perr. & Guill. | Ranunculaceae | THB-082 | Togo | Climber | Evil eye | Leaf | Smelling fresh leaves to the patient | Nasal |
|  |  |  |  |  |  |  | Tying leaves with cloth then put on neck like a necklace | Tie on |
| *Clematis simensis* Fresen. | Ranunculaceae | THB-043 | Turaa | Climber | Herpes labile | Leaf | Washing a diseased part with crushed fresh leaves | Skin |
| *Cleome gynandra* L. | Capparidaceae | THB-035 | Xaaraa | Herb | Diarrhoea | Seed | Roasted seed of the plant pounded then the powder mixed with milk to drink | Oral |

Appendix 1. Continued…

| **Scientific name** | **Family** | **Voucher specimen no.** | **Local name** | **Habit** | **Application** | **Part used** | **Method of preparation** | **Administration route** |
| --- | --- | --- | --- | --- | --- | --- | --- | --- |
| *Clerodendrum umbellatum* Poir. | Lamiaceae | THB-089 | Boye bolleto | Climber | Constipation | Leaf | Well dried fresh leaf using fire pounded and homogenized in water to drink | Oral |
|  |  |  |  |  | Abdominal cramp, diarrhea | Leaf | Crushed fresh leaves homogenized in water to drink | Oral |
| *Commelina latifolia* Hochst. Ex A. Rich | Commelinaceae | THB-045 | Dal77ishaa | Herb | Tenia corporis | Stem | Crushed fresh stem concocted with leaf of *Guizotia schimperi* then soak the diseased part | Skin |
| *Conyza bonariensis* (L.) Cronq. | Asteraceae | THB-014 | Cam77ashaa | Herb | - | - | - | - |
| *Cordia africana* Lam. | Boraginaceae | THB-166 | Mokothaa | Tree | Wound | Leaf | Pounding leaf dried on fire then putting the powder on the wound | Skin |
| *Crossandra nilotica* Oliver | Acanthaceae | THB-033 | Kawo maata | Shrubby herb | Abdominal cramp | Leaf | Crushed fresh leaves serves to drink | Oral |

Appendix 1. Continued…

| **Scientific name** | **Family** | **Voucher specimen no.** | **Local name** | **Habit** | **Application** | **part used** | **Method of preparation** | **Administration route** |
| --- | --- | --- | --- | --- | --- | --- | --- | --- |
| *Crotalaria hyssopifolia* Klotzsch | Fabaceae | THB-137 | Sisebo | Herb | Snake bite | Root | Crushed Fresh root homogenized in water to drink | Oral |
|  |  |  |  |  |  | Root | Chewing fresh root | Oral |
|  |  |  |  |  |  | Whole plant | Smoke bath using dried plant | Nasal |
| *Croton macrostachyus* Del. | Euphorbiaceae | THB-214 | Ankkaa | Tree | Tenia corporis | Leaf | Sap from fresh leaf is smeared on the diseased part | Skin |
|  |  |  |  |  | Wound | Leaf | Sap from fresh leaf is smeared on the cut | Skin |
|  |  |  |  |  |  | Leaf | Dried older leaves are pounded the powder applied on the wound | Skin |
|  |  |  |  |  |  | Leaf | The juice of fresh crushed leaves drop on the wound | Skin |
| *Cuscuta campestris* Yuncker | Cuscutaceae | THB-067 | Melmaalo | Herb | Abdominal Tumor, intestinal parasite | Above ground | Crushed fresh above ground part homogenized in water and mixed with honey to drink | Oral |
| *Cymbopogon citratus* (DC.) Stapf.* | Poaceae | THB-018 | Gattaa | Herb | Influenza | Leaf | A patient will drink decocted leaf mixed with butter | Oral |

Appendix 1. Continued…

| **Scientific name** | **Family** | **Voucher specimen no.** | **Local name** | **Habit** | **Application** | **part used** | **Method of preparation** | **Administration route** |
| --- | --- | --- | --- | --- | --- | --- | --- | --- |
| *Cynoglossum coeruleum* Hochst. ex A. DC. | Boraginaceae | THB-039 | Shidiyaa | Herb | Amoeba | Seed | Roasted seed pounded then eating the powder with food | Oral |
|  |  |  |  |  | Herpes labile | Leaf | Crushed fresh leaves homogenized in water to wash a diseased part | Skin |
| *Cyprus fischerianus* A. Rich.* | Cyperaceae | THB-004 | Bidaaraa | Herb | Abdominal cramp of infants | Root | Crushed fresh root homogenized in water to wash a diseased part | Oral |
| *Dalbergia lactea* Vatke | Fabaceae | THB-064 | Bitbbittoo | Shrub | Evil eye | Leaf | Crushed fresh leaf homogenized in water to wash patient’s body | Skin |
| *Desmodium adscendens* (Sw.) DC. | Fabaceae | THB-040 | Umbbiyaa | Herb | Tinea capitis | Leaf | The powder of dry pounded leaves serves to apply on the diseased part | Skin |
| *Desmodium velutinum* (Willd.) DC. | Fabaceae | THB-163 | Qirccantte dhale | Herb | Snake bite | Root | Crushed Fresh root homogenized in water to drink or chewing fresh root | Oral |
| *Dichrocephala integrifolia* (L. f.) O. Kuntze | Asteraceae | THB-054 | Zea | Herb | Herpes labile | Leaf | Washing face with fresh crushed leaf which is homogenized in water | Skin |
| *Dicliptera laxata* C.B.Clarke* | Acanthacea | THB-021 | Togaa | Herb | Fever | Leaf | Decocted fresh leaf serves for steam bath | Nasal |

Appendix 1. Continued…

| **Scientific name** | **Family** | **Voucher specimen no.** | **Local name** | **Habit** | **Application** | **part used** | **Method of preparation** | **Administration route** |
| --- | --- | --- | --- | --- | --- | --- | --- | --- |
| *Dissotis canescens* (Graham) Hook. f. | Melastomataceae | THB-080 | Zo7o mitha | Herb | Wound | Leaf | Drying fresh leaf then the powder put on the wound | Skin |
|  |  |  |  |  | Abdominal cramp | Leaf | Crushed fresh leaves homogenized in water to drink | Oral |
|  |  |  |  |  | Snake bite | Whole plant | Smoke bath using dry plant | Nasal |
|  |  |  |  |  | Evil eye | Leaf | Crushed fresh leaf put in nose and tie leaf on neck like necklace | Nasal & tie on |
| *Dombeya torrida* (J. F. Gmel.) P. Bamps | Steculaceae | THB-061 | Boshka | Tree | Snake bite | Root | Fresh root decoction serves to wash the bite area | Skin |
| *Dracaena steudneri* Engl. | Dracaenaceae | THB-083 | Ilallaa | Tree | Snake bite | Root | Chewing dried or fresh root | Oral |
| *Dyschoriste multicaulis* (A. Rich.) O. Kuntze | Acanthaceae | THB-146 | Goppe dhaliyaa | Herb | Snake bite | Root | Chewing fresh root then the residue put on the bite | Oral & Skin |
| *Echinops kebericho* Mesfin | Asteraceae | - | Burssaa | Herb | To maintain good health condition | Root | Pounded dry root is mixed with coffee (prepared from matured leaf coffee leaf) | Oral |

Appendix 1. Continued…

| **Scientific name** | **Family** | **Voucher specimen no.** | **Local name** | **Habit** | **Application** | **part used** | **Method of preparation** | **Administration route** |
| --- | --- | --- | --- | --- | --- | --- | --- | --- |
| *Erythrina abyssinica* Lam. ex DC.* | Fabaceae | THB-164 | Borttuwaa | Tree | Abdominal distention, abdominal cramp | Bark | Crushed fresh bark is homogenized in water to drink | Oral |
| *Erythrina brucei* Schweinf.* | Fabaceae | THB-036 | Borttuwaa | Tree | Vomit with diarrhoea, abdominal distention | Bark | Fresh inner bark crushed then homogenized in water to drink | Oral |
| *Eucalyptus camaldulensis* Dehnh.* | Myrtaceae | THB-116 | Barzzaafiya | Tree | Cough | Leaf | Drinking decocted newly emerging leaves | Oral |
|  |  |  |  |  |  |  | Steam bath of decocted fresh leaves | Nasal |
| *Euphorbia schimperiana* Scheele | Euphorbiaceae | THB-068 | Maaro/matsote | Herb | Evil eye | Leaf | Crushed fresh leaf put in nose | Nasal |
|  |  |  |  |  |  | Aboveground | Smelling any fresh plant part | Nasal |
| *Ficus sur* Forssk. | Moraceae | THB-201 | Boo7e | Tree | Wound | Bark | Pounding dried inner bark then put the powder on wound | Skin |

Appendix 1. Continued…

| **Scientific name** | **Family** | **Voucher specimen no.** | **Local name** | **Habit** | **Application** | **part used** | **Method of preparation** | **Administration route** |
| --- | --- | --- | --- | --- | --- | --- | --- | --- |
| *Ficus thonningii* Blume | Moraceae | THB-010 | Shaynaa | Tree | Snakebite | Leaf | The patient served with crushed fresh leaf homogenized in water | Oral |
|  |  |  |  |  |  |  | To prevent allergy the residue smoke bathed | Nasal |
|  |  |  |  |  |  |  | On the next day fresh root of *Kyllinga bulbosa* crushed and homogenized in water for the patient to drink and the residue serves for smoke bath | Oral & nasal |
|  |  |  |  |  |  |  | On the 3^rd^ day crushed concoction of *Oldenlandia goreensis* & *Achyranthes aspera* homogenized in water to drink | Oral |
| *Gardenia ternifolia* Schumach. & Thonn. Subsp. ternifolia | Rubiaceae | THB-072 | Gembbeella | Tree | Snakebite | Root | Washing the bite with decocted root | Skin |
| *Gnidia glauca* (Fresen.) Gilg | Thymeliaceae | THB-056 | Miigiraa | Shrub | Liver disease | Root | Crushed fresh root homogenized in water to drink | Oral |
| *Guizotia schimperi* Sch. Bip. ex Walp. | Asteraceae | THB-044 | Qodhuwaa | Herb | - | - | - | - |

Appendix 1. Continued…

| **Scientific name** | **Family** | **Voucher specimen no.** | **Local name** | **Habit** | **Application** | **part used** | **Method of preparation** | **Administration route** |
| --- | --- | --- | --- | --- | --- | --- | --- | --- |
| *Haumaniastrum villosum* (Benth.) A. J. Paton | Lamiaceae | THB-050 | Wal7o dhale/ Goppe dhaliyaa | Herb | Snake bite | Fruit | Chewing fruit of a plant | Oral |
|  |  |  |  |  |  | Root | Chewing fresh root then soak the bite with the residue | Oral & Skin |
|  |  |  |  |  |  | Whole plant | Smoke bath of the whole plant | Nasal |
| *Indigofera arrecta* Hochst. ex A. Rich. | Fabaceae | THB-017 | Wuswussiyaa | Shrub | Abdominal cramp | Root | A patient drink crushed fresh homogenized root or chew fresh root | Oral |
| *Indigofera emarginella* Steud. ex A. Rich. | Fabaceae | THB-076 | Xawulaa | Shrub | Snake bite | Root | Crushed or pounded fresh or dried root homogenized in water to drink | Oral |
| *Indigofera garckeana* Vatke | Fabaceae | THB-078 | Aamee | Shrub | Snake bite | Root | Crushed fresh root homogenized in water | Oral |
|  |  |  |  |  |  |  | Crushed fresh root put on the bite | Skin |
| *Indigofera spicata* Forssk. | Fabaceae | THB-003 | Danggarssaa dhuure | Herb | Diarrhoea & abdominal cramp | Root | Crushed fresh root homogenized in water to drink or chewing fresh root | Oral |

Appendix 1. Continued…

| **Scientific name** | **Family** | **Voucher specimen no.** | **Local name** | **Habit** | **Application** | **part used** | **Method of preparation** | **Administration route** |
| --- | --- | --- | --- | --- | --- | --- | --- | --- |
| *Indigofera zenkeri* Bak. f. | Fabaceae | THB-103 | Geta amme dhale | Herb | Snake bite | Root | Chewing fresh root then soak or wash the bite area with the residue | Oral & Skin |
|  |  |  |  |  |  | Stem & Leaf | Sun dried stem and leaf serve for smoke bath | Nasal |
| *Isodon ramosissimus* (Hook. f.) Codd | Lamiaceae | THB-088 | Michche dhaliyaa | Herb | Herpes labile | Leaf | Soaking a diseased part with crushed fresh leaf | Skin |
| *Ipomoea purpurea* (L.) Roth. | Convolvulaceae | THB-025 | Turaa | Twiner | Diarrhoea | Leaf | Crushed fresh leaf homogenized in water to drink | Oral |
| *Justicia betonica* L. | Acanthaceae | THB-085 | Goppe dhaliyaa | Herb | Snake bite | Root | Smoke bath | Nasal |
|  |  |  |  |  | Arthritis | Above ground | Smoke bath | Nasal |
| *Kyllinga bulbosa* Vahl. | Cyperaceae | THB-009 | Dhiiringgiya | Herb | - | - | - | - |
| *Lactuca inermis* Forssk. | Asteraceae | THB-060 | Goppe dhaliyaa | Herb | Snake bite | Root | Crushed fresh root homogenized in water to drink, chewing fresh root or root decoction serves to wash the bite area | Oral & Skin |
| *Laggera pterodonata (*DC.) Oliv. | Asteraceae | THB-145 | Haso dhalee | Herb | Snake bite | Root | Chewing fresh root then use the residue to wash the bite | Oral & Skin |

Appendix 1. Continued…

| **Scientific name** | **Family** | **Voucher specimen no.** | **Local name** | **Habit** | **Application** | **part used** | **Method of preparation** | **Administration route** |
| --- | --- | --- | --- | --- | --- | --- | --- | --- |
| *Lantana ukambensis* (Vatke) Verdc. | Verbenaceae | THB-102 | Michmele | Shrub | Abdominal cramp, Diarrhoea | Leaf | Crushed fresh leaf homogenized in water to drink | Oral |
| *Lantana trifolia* L. | Verbenaceae | THB-069 | Michmele | Shrub | Amoeba | Root | Crushed fresh root homogenized in water | Oral |
|  |  |  |  |  | Amoeba | Seed | Chewing seed | Oral |
| *Leonotis ocymifolia* (Burm. f.) Iwarsson var. *raineriana* (Vis.) Iwarsson | Lamiaceae | THB-148 | Eesso | Shrub | Snake bite | Root | Chewing fresh root the soak the bite with the residue | Oral & Skin |
| *Lepidium sativum* L.* | Brassicaceae | THB-086 | Silppaa | Herb | - | - | - | - |
| *Leucas stachydiformis* (Hochst. ex Benth.) Briq. | Lamiaceae | THB-059 | Oythaadhalee | Herb | Apetizer | Leaf | Crushed fresh leaf homogenized in water to drink | Oral |
| *Leucas deflexa* Hook. f. | Lamiaceae | THB-071 | Bikinddo | Herb | Headache with fever | Leaf & Fruit (Seed) | Smelling or eating with pounded plant part | Nasal & Oral |

Appendix 1. Continued…

| **Scientific name** | **Family** | **Voucher specimen no.** | **Local name** | **Habit** | **Application** | **part used** | **Method of preparation** | **Administration route** |
| --- | --- | --- | --- | --- | --- | --- | --- | --- |
| *Lysimachia ruhmeriana* Vatke | Primulaceae | THB-065 | Corqqaa | Herb | Malaria | Root | The juice of crushed fresh root drop in nose | Nasal |
|  |  |  |  |  | To speed up delivery | Leaf | Crushed fresh leaf homogenized in water to drink | Oral |
|  |  |  |  |  | To expel retained placenta | Root | Crushed fresh root homogenized in water to drink | Oral |
|  |  |  |  |  | Evil eye | Stem | Smelling fresh stem then tie it on neck like a necklace | Nasal & tieing on |
|  |  |  |  |  | Snake bite | Whole plant or either of the parts | Drinking decocted fresh plant/plant parts | Oral |
| *Monopsis stellarioides* (Presl) Urb. | Lobeliaceae | THB-106 | Kangadhaa | Herb | Evil spirit | Leaf | Crushed fresh leaves homogenized in water to drink | Oral |
| *Moringa stenopetala* (Bak. f.) Cuf.* | Moringaceae | THB-135 | Halakkuwaa | Tree | Malaria | Bark | Crushed fresh bark homogenized in water to drink | Oral |
|  |  |  |  |  |  | Bark | The juice of crushed fresh bark drop in nose | Nasal |
|  |  |  |  |  |  | Root | Crushed fresh root homogenized in water to drink | Oral |

Appendix 1. Continued…

| **Scientific name** | **Family** | **Voucher specimen no.** | **Local name** | **Habit** | **Application** | **part used** | **Method of preparation** | **Administration route** |
| --- | --- | --- | --- | --- | --- | --- | --- | --- |
| *Mucuna melanocarpa* Hochst. ex. A. Rich. | Fabaceae | THB-34 | Boshaa | Climber | Scabies | Root | Soaking the diseased part with crushed fresh root wich is mixed with butter | Skin |
| *Musa paradisiaca* L.* | Musaceae | - | Muuziya | Herb | Wound | Fruiting stem | Sap from the stem smeared on the wound | Skin |
| *Nigella sativa* L. | Ranunculaceae | - | Karetha sawo | Herb | Intestinal parasite & Diarrhoea | Seed | Concoct dry pounded seed with pounded dry *Brassica juncea* seed and *Echinops kebericho* root then the powder mixed with butter to drink | Oral |
| *Ocimum americanum* L. | Lamiaceae | THB-020 | Deeshsha dunkkiya | Herb | To keep infants healthy | Fruit | Decoction of fresh fruit then homogenizing in water to drink | Oral |
|  |  |  |  |  | Ascariasis | Leaf | Crushed fresh leaf homogenized in water to drink | Oral |
|  |  |  |  |  | Cough | Fruit & Leaf | Drinking fresh decoction mixing with butter | Oral |
|  |  |  |  |  |  | Above ground part | Drinking fresh decoction of any aboveground part mixing with butter | Oral |

Appendix 1. Continued…

| **Scientific name** | **Family** | **Voucher specimen no.** | **Local name** | **Habit** | **Application** | **part used** | **Method of preparation** | **Administration route** |
| --- | --- | --- | --- | --- | --- | --- | --- | --- |
| *Ocimum basilicum* Var. thyrsiflorum (L.) Benth.* | Lamiaceae | THB-028 | Maatha dunkkiya | Herb | Cough (infants) | Fruit (seed) | 1^st^ day The infant will drink decocted fresh fruit (seed) which is mixed with butter  2^nd^ day The infant will drink decocted *Allium cepa* mixed with butter  3^rd^ day The infant will drink decocted *Artemisia afra* mixed with butter | Oral |
| *Ocimum gratissimum* L. | Lamiaceae | THB-131 | Gulo | Shrub | Vomit with fever, Abdominal cramp | Leaf | Crushed fresh leaf homogenized in water to drink | Oral |
| *Ocimum lamiifolium* Hochst. ex Benth.* | Lamiaceae | THB-037 | Daammakasiya | Shrub | Ascariasis, diarrhoea | Leaf | Crushed fresh leaves homogenized in water to drink | Oral |
|  |  |  |  |  | Eye disese | Leaf | Rubbing or putting heated leaf on eye (fresh leaf put on fire) | Eye |
|  |  |  |  |  |  |  | Crushed fresh leaf homogenized in water to wash a diseased part | Skin |
| *Oldenlandia goreensis* (DC.) Summerh. | Rubiaceae | THB-008 | Denqqe | Herb | - | - | - | - |

Appendix 1. Continued…

| **Scientific name** | **Family** | **Voucher specimen no.** | **Local name** | **Habit** | **Application** | **part used** | **Method of preparation** | **Administration route** |
| --- | --- | --- | --- | --- | --- | --- | --- | --- |
| *Oxalis corniculata* L. | Oxalidaceae | THB-049 | Shumecho | Herb | Wound | Leaf | Wash (soak) a wound with fresh crushed leaf | Skin |
| *Paullinia pinnata* L. | Sapindaceae | THB-063 | Qeraa | Climber | Tonsilites | Any part | Crushed any available fresh part of the plant homogenized in water to drink | Oral |
| *Pentas lanceolata* (Forssk.) Defl. Subsp. *quartiniana* (A. Rich.) Verdc. | Rubiaceae | THB-077 | Mithaa | Herb | Snake bite | Leaf | Smoke bath of sun dried leaf pounded for smoke bath | Nasal |
|  |  |  |  |  |  | Leaf | Soaking the whole body with crushed fresh leaf | Skin |
|  |  |  |  |  |  | Whole plant | Chewing any fresh parts of the plant | Oral |
|  |  |  |  |  |  | Root | Smoke bath of root | Nasal |
|  |  |  |  |  |  |  | Chewing fresh root then soaking the bite area with the residue | Oral & Skin |
|  |  |  |  |  |  |  | Crushed fresh root put on the bite | Skin |
| *Phyllanthus sepialis* Muell. Arg. | Euphorbiaceae | THB-055 | Cadhdho | Shrub | Snake bite | Root, Stem or Leaf | Any part of the plant crushed and homogenized in water to drink | Oral |

Appendix 1. Continued…

| **Scientific name** | **Family** | **Voucher specimen no.** | **Local name** | **Habit** | **Application** | **part used** | **Method of preparation** | **Administration route** |
| --- | --- | --- | --- | --- | --- | --- | --- | --- |
| *Phytolacca dodecandra* L’Herit | Phytolaccaceae | THB-181 | Ancciicaa | Shrub | Gonorrhea, Abortion, Liver disease | Root | A patient allowed to drink a juice of fresh crushed root which is homogenized in water or not | Oral |
| *Piper capense* L. F. | Piperaceae | - | Tunjaa | Shrubby herb | Abdominal cramp | Seed | Pounded dry seed homogenized in water to drink | Oral |
| *Pittosporum viridiflorum* Del. | Pittosporaceae | THB-052 | Mittishi7aa | Tree | Ascariasis | Bark | Smelling fresh crushed bark (inner part) | Nasal |
| *Plectranthus lanuginosus* (Hochst. ex Benth.) Agnew* | Lamiaceae | THB-048 | Bunthadhdhiyaa | Herb | Tapeworm | Whole plant | Crushed fresh plant homogenized in water to drink | Oral |
|  |  |  |  |  | Athletics foot | Leaf | Soak foot with crushed fresh leaf | Skin |
| *Plectranthus ornatus* Codd* | Lamiaceae | THB-057 | Disaa | Herb | Abdominal cramp | Root | Drinking a decocted fresh root mixing with butter | Oral |
| *Polygala sadebeckiana* Gurke | Polygalaceae | THB-081 | Goppe dhaliyaa | Herb | Liver disease, Abdominal distention, Snake bite | Root | Crushed fresh root homogenized in water to drink | Oral |
|  |  |  |  |  | Snake bite |  | Chewing fresh root | Oral |

Appendix 1. Continued…

| **Scientific name** | **Family** | **Voucher specimen no.** | **Local name** | **Habit** | **Application** | **part used** | **Method of preparation** | **Administration route** |
| --- | --- | --- | --- | --- | --- | --- | --- | --- |
| *Pterolobium stellatum* (Forssk.) Brenan | Fabaceae | THB-074 | Gom77ore | Climbing shrub | Snake bite | Root | Crushed or pounded fresh or dried root homogenized in water to drink | Oral |
|  |  |  |  |  |  |  | Wash the bite with crushed fresh root | Skin |
| *Ricinus communis* L. | Euphorbiaceae | THB-202 | Xeemaa | Tree | Wound | leaf | Sap from fresh leaves smeared on the wound | Skin |
| *Rumex nepalensis* Spreng. | Polygonaceae | THB-005 | Zanxxallaa | Herb | Abdominal cramp | Root | Crushed fresh root homogenized in water to drink or chewing fresh root | Oral |
| *Ruta chalepensis* L.* | Rutaceae | THB-127 | Xalotiyaa | Herb | Abdominal cramp, diarrhoea of infants | Leaf | Drinking crushed fresh leaf homogenizing with water | Oral |
|  |  |  |  |  | Vomit with diarrhea in infants | Leaf | Crushed fresh leaf homogenizing with water to drink | Oral |
| *Sanicula elata* Buch.-Ham. Ex D. Don. | Apiaceae | THB-147 | Keretha shosha dhaliyaa | Herb | Snake bite | Root | Chewing fresh root then put the residue on the bite | Oral & skin |

Appendix 1. Continued…

| **Scientific name** | **Family** | **Voucher specimen no.** | **Local name** | **Habit** | **Application** | **part used** | **Method of preparation** | **Administration route** |
| --- | --- | --- | --- | --- | --- | --- | --- | --- |
| *Sapium ellipticum* (Krauss) Pax | Euphorbiaceae | THB-047 | Wesaangguwaa | Tree | Abdominal cramp, constipation of infants,  abdominal distention | Leaf | Decocted older leaves mixed with butter to drink | Oral |
|  |  |  |  |  | Diarrhoea | Leaf | Decocted fresh leaf mixed with butter to drink | Oral |
|  |  |  |  |  |  |  | Drinking decocted older leaves with *Allium cepa* | Oral |
|  |  |  |  |  | Snake bite | Root | Crushed fresh root homogenized in water to drink | Oral |
|  |  |  |  |  |  |  | Fresh root decoction serves to wash the bite area | Skin |
| *Setaria megaphylla* (Steud.) Th. Dur. & Schinz | Poaceae | THB-023 | Daawaa | Herb | Snake bite | Leaf | Smell crushed fresh leaf then soak the whole body | Nasal & skin |
|  |  |  |  |  | Abdominal cramp | Leaf | Fresh leaf heat on fire then crush it and smell | Nasal |
| *Sida rhombifolia* L. | Malvaceae | THB-050 | Danddurethaa | Herb | Tumor around neck | Stem | Pieces of stem put on neck like necklace | Tie on |

Appendix 1. Continued…

| **Scientific name** | **Family** | **Voucher specimen no.** | **Local name** | **Habit** | **Application** | **part used** | **Method of preparation** | **Administration route** |
| --- | --- | --- | --- | --- | --- | --- | --- | --- |
| *Solanum incanum* L. | Solonaceae | THB-001 | Buluwaa | Shrub | Abdominal cramp | Root | Chewing fresh root | Oral |
| *Sonchus bipontini* Asch. | Asteraceae | THB-101 | Goppe dhaliyaa | Herb | Snake bite | Root  Leaf & Stem | Chewing fresh root | Oral |
|  |  |  |  |  |  |  | Sun dried leaf and stem for smoke bath | Nasal |
| *Sonchus oleraceus* L. | Asteraceae | THB-151 | Hupe dhako dhale | Herb | Headache | Leaf | Smelling of fresh leaf after heating the leaf on fire | Nasal |
| *Spermacoce sphaerostigma* (A. Rich.) Vatke | Rubiacea | THB-070 | Suufiya | Herb | Herpes labile | Leaf | Crushed fresh leaf homogenized in water to wash diseased part | Skin |
| *Stephania abyssinica* (Dillon & A. Rich.) Walp. | Menispermaceae | THB-022 | Xoussa na7aa haluwaa/Gosha dhale | Twiner | To keep infants healthy | Leaf | The infant will drink fresh crushed leaf which is homogenized in water | Oral |
|  |  |  |  |  | Evil spirit | Leaf | Smelling fresh crushed leaf then tie on neck like a necklace | Nasal & tie on |
| *Terminalia schimperiana* Hochst. | Combretaceae | THB-090 | Ambee | Tree | Abdominal cramp, Diarrhoea | Bark | Crushed fresh bark homogenized in water to drink | Oral |
| *Thalictrum rhynchocarpon* Dill. & A. Rich. | Ranunculaceae | THB-042 | Macchiato | Herb | Diarrhoea | Root | Crushed fresh root homogenized in water to drink | Oral |

Appendix 1. Continued…

| **Scientific name** | **Family** | **Voucher specimen no.** | **Local name** | **Habit** | **Application** | **part used** | **Method of preparation** | **Administration route** |
| --- | --- | --- | --- | --- | --- | --- | --- | --- |
| *Thunbergia ruspolii* Lindau | Acanthaceae | THB-084 |  | Herb | Evil eye | Leaf | Fresh crushed leaf homogenized in water to drink | Oral |
|  |  |  |  |  | Snake bite | Root | Fresh crushed root homogenized in water | Oral |
|  |  |  |  |  |  |  | Chewing fresh root | Oral |
|  |  |  |  |  |  |  | Smoke bath | Nasal |
|  |  |  |  |  |  |  | Fresh crushed root put on the bite | Skin |
| *Tragia brevipes* pax | Euphorbiaceae | THB-012 | Kinkkilishuwaa | Twiner | Tumor around neck | Root | The dried root cut in to pieces then put on neck like necklace | Tie on |
|  |  |  |  |  | Snake bite | Stem | Chewing fresh stem then wash (soak) the bite with the residue | Oral & Skin |
| *Tragia doryoides* M. Gilbert | Euphorbiaceae | THB-029 | Turaa | Twiner | Liver disease | Root | Drinking the juice of fresh crushed root | Oral |
| *Vepris dainellii* (pichi-serm.) Kokwaro* | Rutaceae | THB-027 | Cawulaa | Tree | Abdominal cramp | Seed | Chewing dried seed or pounding dried seed then drinking the powder mixing with coffee | Oral |
| *Vernonia amygdalina* Del.* | Asteraceae | THB-139 | Garaa | Shrub | Snake bite | Root | Crushed fresh root homogenized in water to drink  Fresh root decoction serves to wash the bite area | Oral  Skin |

*Plants cultivated in homegardens with medicinal application
